# Identification and Validation of an Autophagy-Related Gene Signature for Prognostic Prediction and Immunotherapy Response in Esophageal Squamous Cell Carcinoma

**DOI:** 10.3390/cancers18030388

**Published:** 2026-01-27

**Authors:** Rui Chen, Xinran Wang, Guanyang Li, Hao Zhang, Fangqiu Fu, Hanlin Zhou

**Affiliations:** 1 Center for Medical Research and Innovation, School of Life Sciences, Human Phenome Institute, Shanghai Pudong Hospital, Fudan University, Shanghai 200438, China; s2025003052@student.pumc.edu.cn (R.C.); 25212030022@m.fudan.edu.cn (X.W.); 22307110227@m.fudan.edu.cn (G.L.); 2 National Central Cancer Registry Office, National Cancer Center, National Clinical Research Center for Cancer, Cancer Hospital, Chinese Academy of Medical Sciences, Peking Union Medical College, Beijing 100021, China; 3 Shenzhen Institute of Advanced Technology, Chinese Academy of Sciences, Shenzhen 518055, China; h.zhang10@siat.ac.cn; 4 Department of Thoracic Surgery, Fudan University Shanghai Cancer Center, Shanghai 200032, China

**Keywords:** esophageal squamous cell carcinoma, autophagy, prognostic model, autophagy-related genes, transcriptomic data, personalized treatment, tumor microenvironment

## Abstract

Purpose: Despite existing signatures, there remains a lack of robust autophagy-based biomarkers validated across multi-omics datasets and independent clinical cohorts in ESCC. The aim of our study was to develop an autophagy-related prognostic model for ESCC. Methods: Using transcriptomic data of ESCC from GEO/TCGA and autophagy-related genes (ARGs) from five autophagy-specific datasets, we identified the intersection between ARGs and tumor-normal differentially expressed genes (DEGs). We constructed a prognostic model using stepwise multivariate Cox regression based on these genes in GSE53625 (*n* = 179), validated in TCGA-ESCC (*n* = 94) through survival analysis and ROC curve, and analyzed the prognostic value of candidate genes in in-house ESCC samples. Results: We successfully established a robust prognostic 4-ARGs model comprising *NBEA*, *CLOCK*, *NLRX1*, and *MAGEA3* (training: *p* < 0.0001, validation: *p* = 0.013). In the in-house ESCC cohort (*n* = 14), *NLRX1* was verified as a reliable prognostic factor for disease-free survival (*p* = 0.043). Significant correlations were observed between signatures and the immune microenvironment, and the model effectively predicted patients’ responses to immunotherapy. Conclusion: We developed a novel 4-ARGs prognostic model and identified *NLRX1* as a potential autophagy-dependent biomarker. These findings underscore its utility as a valuable tool for prognosis, risk stratification, and therapy guidance in ESCC.

## Simple Summary

Esophageal squamous cell carcinoma (ESCC) accounts for >90% of all esophageal cancer cases in China. While autophagy exerts dual roles in ESCC, its gene-level prognostic value remains undefined. The aim of our study was to develop an autophagy-related prognostic model for ESCC. We integrated autophagy-related genes (ARGs) with ESCC transcriptomic data to construct a robust 4-ARG model with multi-cohort validation (comprising *NBEA*, *CLOCK*, *NLRX1*, and *MAGEA3*) via stepwise multivariate Cox regression. Significant survival differences were observed between high- and low-risk groups stratified by the model (*p* < 0.001). Additionally, the signature correlated significantly with the immune microenvironment and predicted patients’ responses to immunotherapy. In an in-house ESCC cohort (*n* = 14), *NLRX1* was verified as a reliable prognostic factor for disease-free survival (*p* = 0.043). Our findings demonstrate that the 4-ARG model enables ESCC prognosis prediction and identifies *NLRX1* as a stable biomarker, providing a theoretical basis for personalized risk stratification and therapeutic decision-making in ESCC patients.

## 1. Introduction

Esophageal cancer is a highly prevalent malignant tumor of the digestive tract, featuring substantial morbidity and mortality. It is mainly classified into two histological subtypes: esophageal adenocarcinoma (EAC) and esophageal squamous cell carcinoma (ESCC) [1]. Data from 2022 indicates that esophageal cancer is among the top five leading causes of cancer-associated deaths in China, contributing to roughly 187,500 fatalities [2]. ESCC represents the dominant histological subtype in China, with notably higher incidence and mortality rates than those in other nations [3]. Despite advances in diagnostic and therapeutic techniques, ESCC continues to have a poor prognosis, with an overall 5-year survival rate of only 15% to 25% [1,4]. Early diagnosis and in-depth exploration of the genetic and molecular mechanisms driving ESCC are crucial for advancing diagnostic accuracy, therapeutic efficacy, and patient prognosis.

Autophagy is a critical and complex homeostatic process that plays a role in numerous biological pathways. During autophagy, double-membrane vesicles called autophagosomes engulf cellular proteins and organelles, delivering them to lysosomes for degradation. The autophagy process is regulated by autophagy-related genes (ARGs) [5]. Autophagy is often regarded as a double-edged sword in cancer [6]. Autophagy can either suppress or facilitate tumor progression, depending on tumor type, clinical stage, genetic profile, and therapeutic strategy [7]. For example, in precancerous lesions, accumulating evidence suggests that boosting autophagy may hinder carcinogenesis [8]. Conversely, in advanced malignancies, both autophagy promotion and inhibition have been proposed as potential therapeutic approaches [9]. The dual role of autophagy in ESCC leads to prognostic uncertainty. Many steps in the autophagy pathway represent potential druggable targets, offering opportunities to either enhance or inhibit autophagy. Several research results have demonstrated the potential of autophagy inhibitors in cancer treatment [10,11,12]. Despite advances in treatment, ESCC patients still face poor prognosis due to lack of reliable prognostic biomarkers for risk stratification and personalized therapy guidance. Although autophagy has been implicated in chemotherapy resistance in ESCC [13,14], its precise role, prognostic value and clinical significance in ESCC patients remain unclear. Therefore, identifying molecular biomarkers based on autophagy is crucial for improving prognosis prediction and therapeutic strategies in ESCC.

Large-scale datasets (GEO/TCGA) provide sufficient sample size and comprehensive transcriptomic information to identify robust gene signatures, overcoming the limitations of small sample studies. Compared with single biomarkers, multi-gene signatures can capture complex biological processes involved in ESCC progression, achieving higher predictive accuracy and clinical applicability in ESCC. In the study, we conducted a comprehensive analysis of ESCC transcriptomic datasets to screen 140 differentially expressed autophagy-related genes (ARGs). Utilizing machine learning techniques, we further established a 4-ARG prognostic model incorporating *NBEA*, *CLOCK*, *NLRX1*, and *MAGEA3*. This model’s predictive capability was validated in multiple independent cohorts, confirming its robust prognostic significance. *NLRX1* was confirmed as a reliable prognostic factor using molecular biological experiments among in-house ESCC samples. Furthermore, the model effectively predicted immunotherapy response and pan-cancer prognostic value. Our results may provide valuable insights for personalized treatment and prognosis of ESCC patients.

## 2. Materials and Methods

### 2.1. Data Collection

A total of 1357 autophagy-related genes (ARGs) were extracted by integrating data from five databases (Supplementary Table S1): 232 from the Human Autophagy Database (HADb, https://autophagy.lu/v1/, accessed on 27 March 2024), 358 from the GOBP_REGULATION_OF_AUTOPHAGY gene set in MSigDB v7.1 [15], 550 from the Proteins with autophagy information gene set in Index-Home-Human Autophagy Moderator Database (HAMdb) [16], 587 from ncRDeathDB database [17], 742 from Autophagy Database [18]. We acquired transcriptomic datasets (including both RNA-seq and scRNA-seq) and associated clinical metadata from the Gene Expression Omnibus (GEO) (https://www.ncbi.nlm.nih.gov/geo, accessed on 8 December 2024) and The Cancer Genome Atlas (TCGA) (https://www.cancer.gov/tcga/, accessed on 8 December 2024). The study cohort encompassed six specific datasets: TCGA-ESCC (*n* = 94), GSE53625 (*n* = 358) [19], GSE160269 (*n* = 64) [20], GSE78220 (*n* = 28) [21], and GSE67501 (*n* = 11) [22], and the IMvigor210 cohort (*n* = 310) [23]. The datasets used and their information are summarized in Table S2. The clinical information of patients in the training set (GSE53625) and validation set (TCGA-ESCC) is summarized in Table S3.

### 2.2. RNA-Seq Expression Data Processing

For genes matched by multiple probes, average expression values were used for microarray data. Quantile normalization was applied to bulk RNA-seq data. RNA-seq gene expression data were log2-transformed and standardized to z-scores. Samples with survival times under one month or incomplete data were excluded to ensure reliability. The GSE53625 dataset was used as the training set, while TCGA-ESCC served as validation sets for assessing the ESCC prognostic model’s accuracy. Furthermore, to evaluate the model’s predictive power regarding sensitivity to immunotherapy, we incorporated data from immune checkpoint inhibitor therapy clinical studies. This included an analysis of metastatic urothelial carcinoma patients treated with anti-PD-L1 treatment (IMvigor210 cohort), melanoma patients receiving anti-PD-1 therapy as documented in GSE78220, and Renal Cell Carcinoma patients treated with anti-PD-L1 therapy (GSE67501).

### 2.3. scRNA-Seq Data Processing

The GSE160269 dataset was processed using Seurat (v4.1.0) [24]. Quality control involved filtering out cells with <500 or >7500 genes and >20% mitochondrial content, and removing genes which were present in fewer than three cells. DoubletFinder (v2.0.4, 8% rate) [25] was used to identify doublets with an expected doublet rate of 8%. Harmony (v1.2.0) [26] was employed to eliminate batch effects. The Seurat package was employed to conduct cell clustering and differential expression profiling. Cluster identities were assigned based on the expression profiles of canonical marker genes. Subsequently, to discriminate between malignant and normal epithelial cells, we utilized the infercnv algorithm (https://github.com/broadinstitute/inferCNV, accessed on 8 December 2024).

### 2.4. Establishment of the Prognostic Model Based on Autophagy-Related Genes

For the development of the ESCC prognostic model, we initially screened the GSE53625 dataset using the “limma” R package (v 3.62.2) [27], isolating 809 genes differentially expressed between tumor and normal tissues (*p* < 0.05, |logFC| > 1). These differentially expressed genes (DEGs) were then cross-referenced with the ARG list. Following this, a univariate Cox regression analysis was executed via the “survival” package [28] to identify candidate genes significantly correlated with overall survival (OS) (*p* < 0.05). A stepwise multivariate Cox regression analysis was subsequently performed using a bidirectional selection process, including back-ward stepwise elimination and forward stepwise selection, to build the prognostic model (*p* < 0.05), implemented via the “stepAIC” function in the “MASS” R package [29]. This resulted in a 4-ARGs prognostic model, with the following calculation formula:

Risk score = 0.45902 × expr (*NBEA*) + 0.77386 × expr (*CLOCK*) − 0.31304 × expr (*NLRX1*) − 0.09516 × expr (*MAGEA3*),

where the coefficients in the formula are derived from the stepwise multivariate Cox regression, and expr (Gene) denotes the expression level of a specific gene.

Using this formula, risk scores for ESCC patients were calculated, and patients were stratified into high-risk (*n* = 89) and low-risk (*n* = 90) groups based on the median risk score.

### 2.5. Validation of the Candidate Genes and the 4-ARGs Model

In the validation phase, we established optimal risk stratification thresholds using the surv_cutpoint function from the “survminer” package [30]. Kaplan–Meier (KM) survival analyses for high- and low-risk subgroups were generated via the “survival” package and visualized using ggsurvplot. To gauge the model’s predictive precision, time-dependent receiver operating characteristic (ROC) curves for 1-, 2-, and 3-year intervals were constructed using the “timeROC” package [31]. The area under the curve (AUC) served as the performance metric, with values approaching 1 indicating superior predictive efficacy.

### 2.6. Construction of a Nomogram

To isolate factors serving as independent predictors of OS in ESCC patients, we performed both univariate and multivariate Cox regression analyses, treating *p* < 0.05 as the significance threshold. We used the R package “rms” [32] to integrate these independent prognostic factors and constructed nomograms for 1, 2, and 3 years’ predictions along with their corresponding calibration plots. The calibration plots were validated through calibration and discrimination. Calibration curves were generated to visually assess the nomogram’s accuracy. A close alignment between the calibration curve and the diagonal reference line denotes optimal consistency between predicted probabilities and observed outcomes.

### 2.7. Gene Set Enrichment Analysis (GSEA)

We investigated the functional implications of DEGs across risk groups by performing enrichment analyses using the enrichGO and enrichKEGG algorithms within the “clusterProfiler” R package [33]. The results were visualized with the “enrichplot” R package (v1.22.0) [34]. Additionally, GSEA was performed using KEGG (C2) and Hallmark (H) gene sets to identify differentially activated pathways and biological processes between the two groups. Pathway significance was defined by meeting three criteria simultaneously: a False Discovery Rate (FDR) < 0.25, a nominal *p*-value < 0.05, and a Normalized Enrichment Score (|NES|) exceeding 1.

### 2.8. Immune Infiltration Analysis

Interactions between the 4-ARG model candidate genes and the tumor immune microenvironment (TIME) were evaluated via the “ESTIMATE” package (v1.0.13/r21) [35], which computed stromal, immune, and aggregate scores. Concurrently, the relative proportions of 22 distinct immune cell subsets were inferred using the CIBERSORT algorithm. Based on linear ν-support vector regression (ν-SVR), CIBERSORT deconvolves relative proportions of immune cell subsets from mixed tissue expression profiles using a reference signature matrix, the LM22 background gene set from the CIBERSORT website (https://cibersort.stanford.edu/, accessed on 8 December 2024) [36]. Additionally, the absolute infiltration levels of eight immune and two stromal cell populations were quantified utilizing the “MCPcounter” R package (v1.2.0) [37]. MCPcounter quantifies absolute abundance of target cells by calculating the log2 geometric mean of cell-type-specific transcriptomic markers that are highly expressed and stably expressed in specific cell populations. To investigate the potential association between the abundance of immune infiltrates and the expression profiles of candidate genes, we computed Pearson correlation coefficients.

### 2.9. Predicting Responses to Immune Checkpoint Inhibitors (ICIs) Using the 4-ARGs Model

Responsiveness to Immune Checkpoint Inhibitors (ICIs) was predicted using the Tumor Immune Dysfunction and Exclusion (TIDE) framework (http://tide.dfci.harvard.edu/, accessed on 8 December 2024) [38]. This platform integrates comprehensive omics data and biomarkers from 188 tumor cohorts, along with results from 12 ICI trials and eight CRISPR screens focused on anti-tumor immunity mechanisms. Patients yielding TIDE scores < 0 were categorized as responders, whereas those with scores > 0 were deemed non-responders. The TIDE algorithm also calculates dysfunction and exclusion scores to evaluate T-cell dysfunction and immune exclusion, respectively. Higher total TIDE scores indicate poorer treatment response and prognosis. We further validated these findings using three external immunotherapy cohorts (IMvigor210, GSE78220, and GSE67501), comparing treatment efficacy between risk groups. Clinical outcomes were classified into four categories: complete response (CR), partial response (PR), progressive disease (PD), and stable disease (SD).

### 2.10. Drug Sensitivity Analysis

The Potential therapeutic agents capable of reversing or inducing specific biological states associated with the identified markers were explored via the Connectivity Map (CMap) database [39,40]. To investigate the potential activity of compounds in CMap database for patients with ESCC, we retrieved DEGs between normal and tumor tissues from ESCC patients in the GSE53625 dataset, and uploaded them to the CMap database for mode-of-action (MoA) analysis. We have selected the top 50 potential compounds with the highest predictive scores, considering them to be the most potentially effective for ESCC, and cataloged their targets and pathways of action.

Drug sensitivity analysis was further performed for high-risk group and low-risk group in GSE53625 using the Genomics of Drug Sensitivity in Cancer (GDSC) database [41] via the “OncoPredict” R package (v1.2) [42]. Chemotherapeutic sensitivity was assessed by constructing a ridge regression model using the calcPhenotype function to predict the half-maximal inhibitory concentration (IC50) for 198 drugs. IC50 value is a concept derived from standard in vitro pharmacology assays. It refers to the drug concentration required to inhibit 50% of the proliferation or viability of in vitro cultured cancer cell lines over a specific period (typically 72 or 96 h). Lower IC50 values signify heightened drug sensitivity. We evaluated the clinical utility of the risk score by comparing projected IC50 values between the two risk groups.

### 2.11. Pan-Cancer Analysis

To test whether the 4-ARGs model and candidate genes have prognostic value for other cancer types, the “TCGAplot” R package [43] was employed to perform pan-cancer analyses in TCGA. The candidate genes’ expression in samples from 33 types of cancer patients was analyzed and visualized using “TCGAplot”. We integrated pan-cancer survival data and expression data in TCGA to analyze the relationship between OS and the 4-ARGs model and candidate genes using univariate Cox regression analysis. The resulting hazard ratio (HR) and *p*-value were visualized using the R package “ggplot2” [44].

### 2.12. Gene Set Variation Analysis (GSVA)

To investigate the relationship between the candidate genes and autophagy-related functions, Pearson correlation coefficients were calculated between the expression levels of the candidate genes and autophagy score in the GSE53625 dataset. Autophagy scores were calculated based on the expression profiles of 1357 autophagy-related genes for each sample using the R package GSVA (version 1.40.1) [45].

### 2.13. Quantitative RT-PCR Validation

Paired primary tumor and normal adjacent tissues (NATs) were prospectively harvested from 14 ESCC patients undergoing surgical resection at the Department of Thoracic Surgery, Fudan University Shanghai Cancer Center (FUSCC). Consistent with established protocols, NATs were collected ≥3 cm from the tumor margin. The study received ethical approval from the FUSCC Review Committee, and informed consent was secured from all participants. Following total RNA extraction, qRT-PCR assays were conducted on a LightCycler 480 II System utilizing the SGExcel FastSYBR Mixture (Sangon, Shanghai, China). Expression levels were normalized to GAPDH via the 2^−ΔΔCT^ method. Primer sequences and cohort details are provided in Supplementary Tables S2 and S4, respectively.

### 2.14. Statistical Analysis

Statistical computations were executed using R software (v4.4.2). Group differences were evaluated via Wilcoxon or *t*-tests, while correlation matrices were analyzed using Spearman or Pearson methods. Survival disparities were assessed using Kaplan–Meier plotting and log-rank testing, with statistical significance established at *p* < 0.05.

## 3. Results

### 3.1. Identification of Autophagy-Related Genes with Differential Expression Patterns in Esophageal Squamous Cell Carcinoma

**Figure 1:**
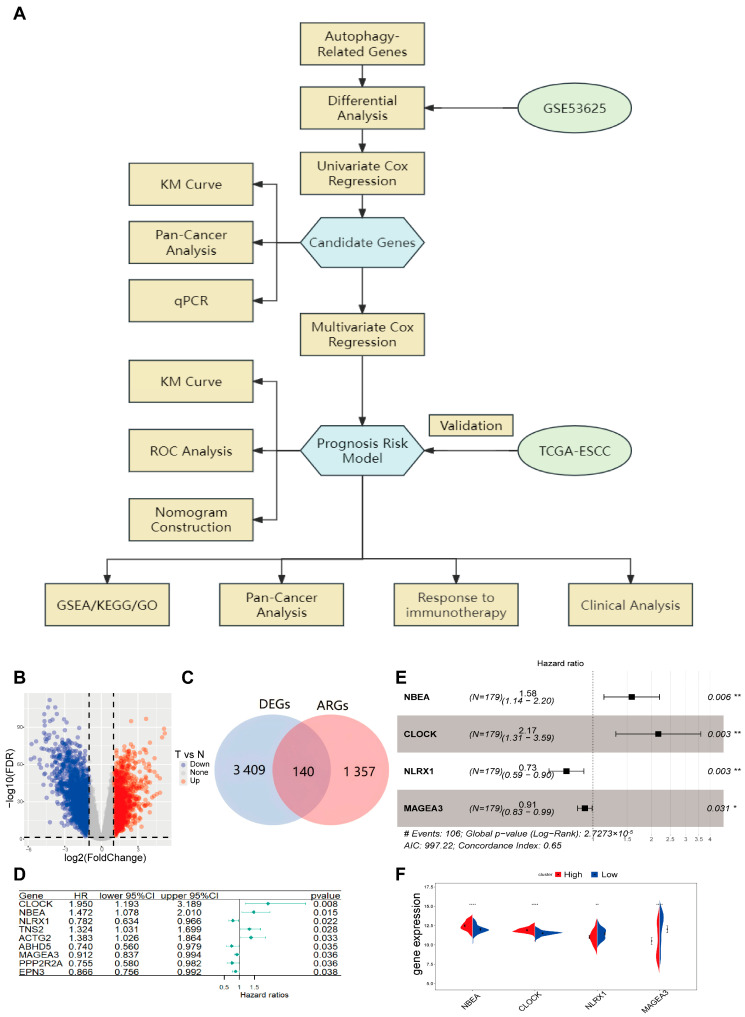
The flow chart of this study and establishment of the prognostic model. (**A**) The flow chart of this study; (**B**) The volcano plot of the DEGs between normal and tumor tissue in GSE53625 (*n* = 179); (**C**) Intersection of ARGs and DEGs; (**D**) Results of univariate Cox regression analysis identified 9 ARGs associated with OS; (**E**) Results of stepwise multivariate Cox regression analysis identified 4 ARGs associated with OS; (**F**) The expression of the 4 candidate genes in high-risk (*n* = 89) and low-risk (*n* = 90) groups in GSE53625 (Wilcox test, * *p* < 0.05; ** *p* < 0.01; **** *p* < 0.0001).

A schematic representation of the comprehensive study design and analytical pipeline is depicted in Figure 1A. After removing duplicate genes from the MSigDB, HADb, HAMdb, ncRDeathDB, and Autophagy databases, a comprehensive autophagy-related gene set comprising 1357 genes was obtained (Supplementary Table S1). The ESCC dataset GSE53625, consisting of microarray data from 179 ESCC tissues and paired normal esophageal tissues, was used to identify differentially expressed autophagy-related genes (de-ARGs). Differentially expressed genes (DEGs) were identified based on the criteria |logFoldChange| > 1 and *p* < 0.05, resulting in 3409 DEGs, including 1985 downregulated and 1424 upregulated genes (Figure 1B). By intersecting the DEG set with the autophagy gene set, 140 de-ARGs were identified (Figure 1C).

### 3.2. Establishment of the Prognostic Model Based on Autophagy-Related Genes

A prognostic model based on de-ARGs was constructed using the GSE53625 dataset. Univariate Initial screening via Cox regression analysis pinpointed nine differentially expressed autophagy-related genes (de-ARGs) with significant prognostic relevance to overall survival (OS) (*p* < 0.05, Figure 1D). To refine this selection into a robust risk model, a stepwise multivariate Cox regression was subsequently executed (Figure 1E). The culminating model incorporated four specific de-ARGs: *NBEA*, *CLOCK*, *NLRX1*, and *MAGEA3*. The risk scoring algorithm for this 4-ARG signature was established as follows:

Risk score = 0.45902 × expr (*NBEA*) + 0.77386 × expr (*CLOCK*) − 0.31304 × expr (*NLRX1*) − 0.09516 × expr (*MAGEA3*),

Using this formula, following the computation of individual risk scores for the ESCC cohort, the median risk score served as the threshold to bifurcate patients into a high-risk group (*n* = 89) and a low-risk group (*n* = 90). In this model, *NBEA* and *CLOCK* were identified as risk factors, while *NLRX1* and *MAGEA3* were protective factors. We observed marked disparities in the expression levels of these four signature genes between the two risk groups. These expression patterns were congruent with their respective coefficients in the risk calculation formula (Figure 1F).

**Figure 2:**
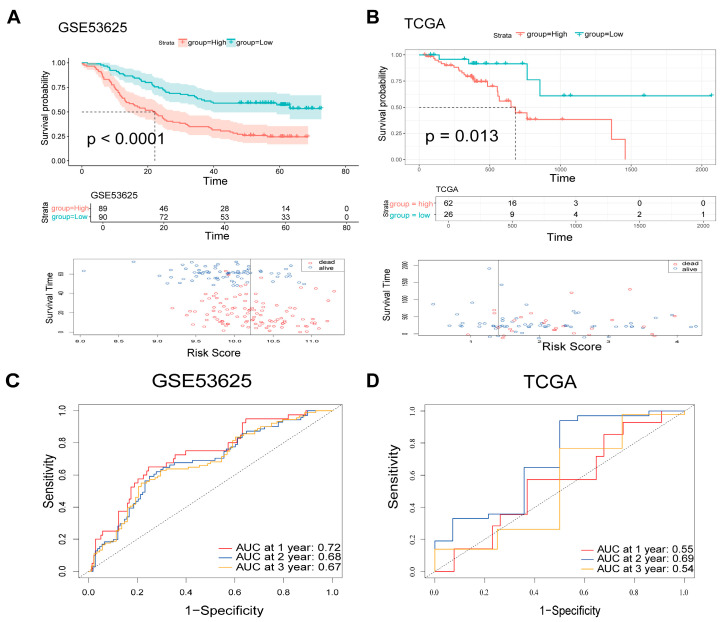
Validation of the 4-ARGs model. The Kaplan–Meier survival analysis and scatter plot for ESCC patients in high- and low-risk group in GSE53625 (*n* = 179) (**A**) and TCGA-ESCC (*n* = 94) (**B**), and the dotted line represents the median survival time.; The time-dependent ROC curve for ESCC patients in the training dataset (GSE53625) (**C**) and validation dataset (TCGA-ESCC) (**D**).

The 4-ARGs model effectively predicted patient prognosis in the GSE53625 dataset. Kaplan–Meier analysis revealed a significant prognostic disparity, where high-risk patients exhibited substantially poorer survival compared to the low-risk group (log-rank test, *p* < 0.0001). The distribution of 4-ARGs model for patients in GSE53625 was shown in Figure 2A. The model demonstrated reliable predictive performance, achieving AUC values of 0.72, 0.68, and 0.67 for 1-, 2-, and 3-year survival, respectively (Figure 2C). Additionally, KM survival analyses for the four individual genes confirmed their prognostic value in GSE53625 (Figure S1A).

### 3.3. Validation of the Candidate Genes and the 4-ARGs Model

To corroborate the generalizability and robustness of the 4-ARG model, we leveraged an external independent cohort (TCGA-ESCC). Consistent with the training set, KM analysis confirmed that high-risk patients in the validation cohort faced significantly poorer survival prospects than those in the low-risk group (log-rank test, *p* < 0.05). Risk distribution and survival outcomes for the validation dataset are shown in Figure 2B. The AUC values for 1-, 2-, and 3-year survival predictions exceeded 0.5, with some values approaching 0.7, further supporting the model’s reliability (Figure 2D). Similarly, the candidate genes showed consistent predictive performance across the validation dataset (Figure S1B).

### 3.4. Identification of Independent Clinical Prognostic Factors and Nomogram Construction

**Figure 3:**
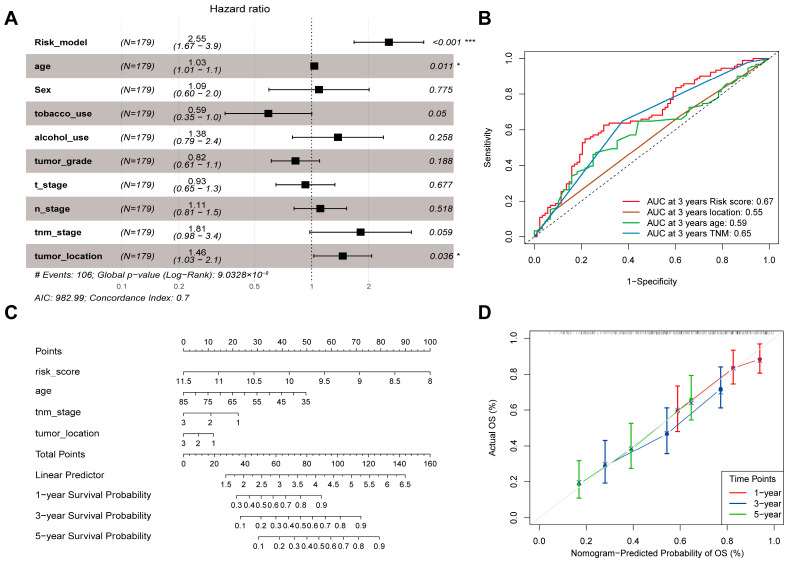
Recognition of independent clinical prognostic factors and nomogram construction. (**A**) The forest plot of multivariate Cox regression analysis based on risk score and clinical factors (GSE53625, *n* = 179) (* *p* < 0.05; *** *p* < 0.001); (**B**) The time-dependent ROC curve for risk score and independent clinical prognosis factors; (**C**) Nomogram constructed by combining clinical factors and the risk score. Tumor-position codes: 1 = lower, 2 = middle, 3 = upper of the esophagus; (**D**) The calibration curves of nomogram for predicting 1-, 2-, and 3-year OS (The dots above the plot represent the distribution of Nomogram-Predicted Probability of OS for all samples).

To identify independent prognostic factors for ESCC, clinical characteristics and the 4-ARGs model were analyzed using multivariate Cox regression. Age, tumor location, and the 4-ARGs model were identified as significant independent prognostic factors, while TNM stage showed borderline significance (*p* = 0.059, Figure 3A). Although TNM stage showed borderline significance (*p* = 0.059 in the multivariate analysis, it was retained in the final nomogram due to its indispensable role in current clinical practice and its biological relevance to patient survival. Receiver operating characteristic (ROC) curve analysis indicated that the 4-ARGs model was the most accurate independent prognostic factor (Figure 3B).

To provide a practical tool for predicting patient survival, a nomogram was constructed based on multivariate Cox regression analysis, incorporating age, tumor location, TNM stage, and the 4-ARGs model (Figure 3C). Scores for each prognostic factor were calculated and summed to predict 1-, 2-, and 3-year overall survival (OS) rates. Calibration curves for the nomogram closely aligned with the ideal prediction curve, indicating excellent agreement between predicted and observed OS rates (Figure 3D).

### 3.5. Analysis of Pathway Differences Between High-Risk and Low-Risk Groups

**Figure 4:**
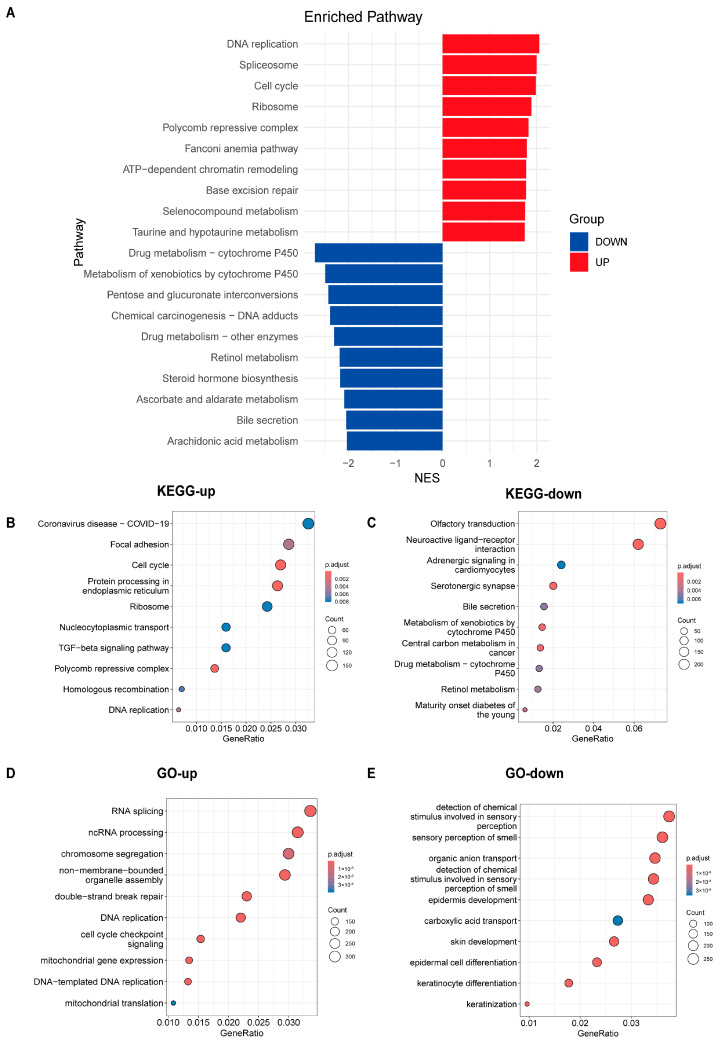
Functional landscape and pathway enrichment associated with the 4-ARG signature (GSE53625, *n* = 179). (**A**) Gene Set Enrichment Analysis (GSEA) highlighting pathways enriched in upregulated versus downregulated genes. (**B**,**C**) KEGG pathway analyses for upregulated and downregulated genes, respectively. (**D**,**E**) GO annotation analyses for upregulated and downregulated genes.

Through differential expression analysis within the GSE53625 dataset, we identified 846 genes distinguishing the high-risk from the low-risk group, comprising 309 upregulated and 537 downregulated transcripts (*p* < 0.05, |logFoldChange| > 1). Biological heterogeneity was explored through Gene Set Enrichment Analysis (GSEA), complemented by KEGG and GO functional annotations (Figure 4A–E).

GSEA revealed that tumor-associated pathways such as “Base excision repair”, “ATP-dependent chromatin remodeling”, “Fanconi anemia pathway”, “Cell cycle” and “DNA replication” were significantly upregulated in the high-risk group, while “Chemical carcinogenesis—DNA adducts” was downregulated (Figure 4A). KEGG analysis showed that upregulated DEGs were enriched in pathways like “Focal adhesion”, “Cell cycle”, “TGF-beta signaling pathway” and “DNA replication” (Figure 4B), while downregulated DEGs were enriched in “Central carbon metabolism in cancer” (Figure 4C). GO analysis further confirmed that upregulated DEGs were associated with processes critical to tumor biology, such as “double-strand break repair,” “DNA replication” and “cell cycle checkpoint signaling” (Figure 4D). The results show that several tumor-related pathways are significantly up- or down-regulated, indicating that the OS difference between the high- and low-risk groups is, at least in part, attributable to alterations in these pathways. Moreover, the different expression level of tumor-associated pathways across the two groups defined by our model indirectly corroborates the model’s capability to effectively stratify patients.

Notably, pathways related to xenobiotic processing, specifically “Drug metabolism—cytochrome P450” and “Metabolism of xenobiotics by cytochrome P450,” appeared significantly suppressed in the high-risk group. The Cytochrome P450 (CYP) superfamily encompasses hemoproteins that are ubiquitous across organisms and are pivotal for catalyzing the Phase I biotransformation of a vast array of endogenous substrates and xenobiotics. In the United States, it is estimated that these enzymes mediate the metabolism of approximately 60% of commonly prescribed pharmacotherapies [46]. Although CYPs inactivate many cytotoxins, they are essential for activating specific prodrugs in chemotherapy [47]. However, inconsistencies in methodology and patient heterogeneity have led to conflicting evidence regarding the precise role of CYPs in drug metabolism [48]. Based on our findings, the 4-ARGs model suggests that patients in the high-risk group may experience reduced efficacy of anticancer drugs and shortened overall survival (OS) due to downregulation of the CYP450 drug metabolism pathway. Previous studies have suggested a potential relationship between autophagy and the cytochrome P450 (CYP) drug metabolism pathway. Specifically, research has demonstrated that the CYP inhibitor SKF-525A induces a significant accumulation of microtubule-associated protein light chain 3 II (LC3-II) in primary rat hepatocytes, indicating a disruption in autophagy. Furthermore, SKF-525A has been shown to increase p62 protein levels and inhibit the fusion of autophagosomes with lysosomes, providing additional evidence that autophagic flux is blocked [49]. Taken together, these suggest that the downregulation of CYP pathways in high-risk patients may impair autophagy and contribute to poor prognosis.

### 3.6. Immune Infiltration Analysis and Relationship Between Candidate Genes and Immunity

**Figure 5:**
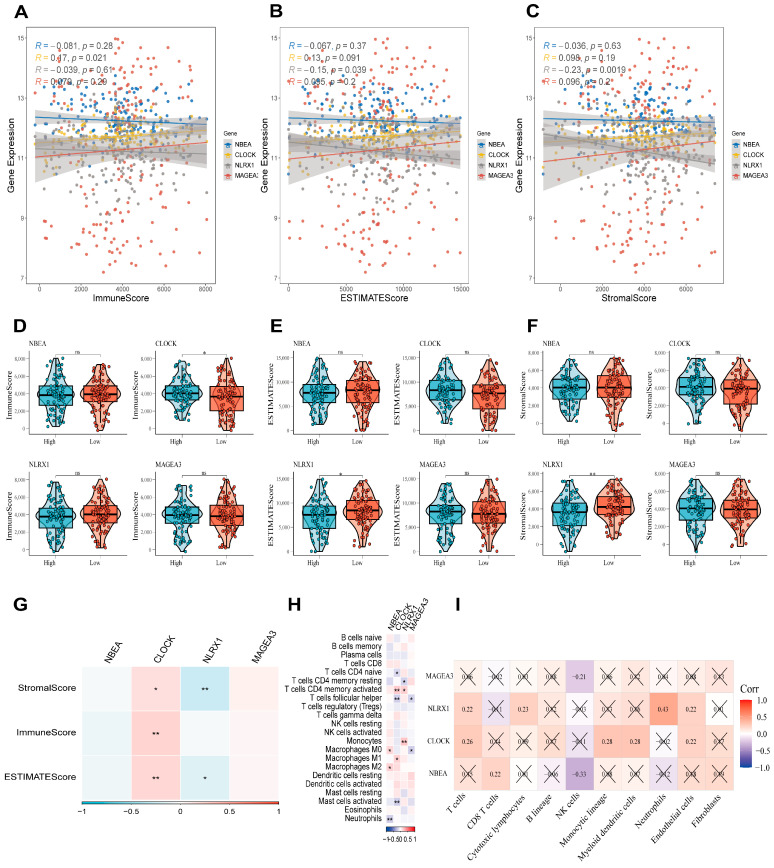
Immune infiltration analysis and relationship between candidate genes and immunity (GSE53625, *n* = 179). (**A**–**C**) Scatter plots depicting linear regression analyses between candidate gene expression and Immune, ESTIMATE, and Stromal scores. (**D**–**G**) Boxplots illustrating the correlation of candidate gene expression with these microenvironmental scores. (**H**) Heatmap showing correlations between candidate gene expression and the proportions of 22 immune cell subsets. (**I**) Correlation matrix between candidate gene expression and the abundance of 8 immune and 2 stromal cell populations (non-significant associations crossed out; * *p* < 0.05; ** *p* < 0.01; ns, not significant).

We further probed the nexus between the four candidate genes and immune microenvironment characteristics within the GSE53625 dataset. Correlation analysis was conducted between gene expression and the three scores (stromal, immune, and ESTIMATE scores). A consistent positive association between the risk-related gene *CLOCK* and the three scores was revealed, whereas the protective gene *NLRX1* displayed an inverse relationship with stromal and ESTIMATE scores (Figure 5G). Subsequent stratification of samples based on median gene expression corroborated these findings, showing significant disparities in scores between high- and low-expression groups (Figure 5D–F). Linear regression analysis further supported these findings (Figure 5A–C).

The cellular composition of the immune microenvironment was deconvoluted using the CIBERSORT algorithm. Distinct associations emerged between the four candidate genes and specific immune cell subsets (Figure 5H). The expression of *NBEA* exhibited a positive correlation with M0/M2 macrophages, whereas a negative correlation was observed with neutrophils. In contrast, *CLOCK* demonstrated positive associations with M1 macrophages and activated CD4 memory T cells, but displayed inverse correlations with naive CD4 T cells, follicular helper T cells, and activated mast cells. *NLRX1* paralleled the abundance of activated CD4 memory T cells and monocytes but was inversely related to resting CD4 memory T cells. *MAGEA3* showed negative correlations with follicular helper T cells and M0 macrophages. Furthermore, quantification via the MCPcounter algorithm revealed that both *CLOCK* and *NLRX1* were positively linked to the abundance of multiple immune and stromal cell types (Figure 5I).

### 3.7. Immunotherapy Response in High- and Low-Risk Groups Defined by the 4-ARGs Model

**Figure 6:**
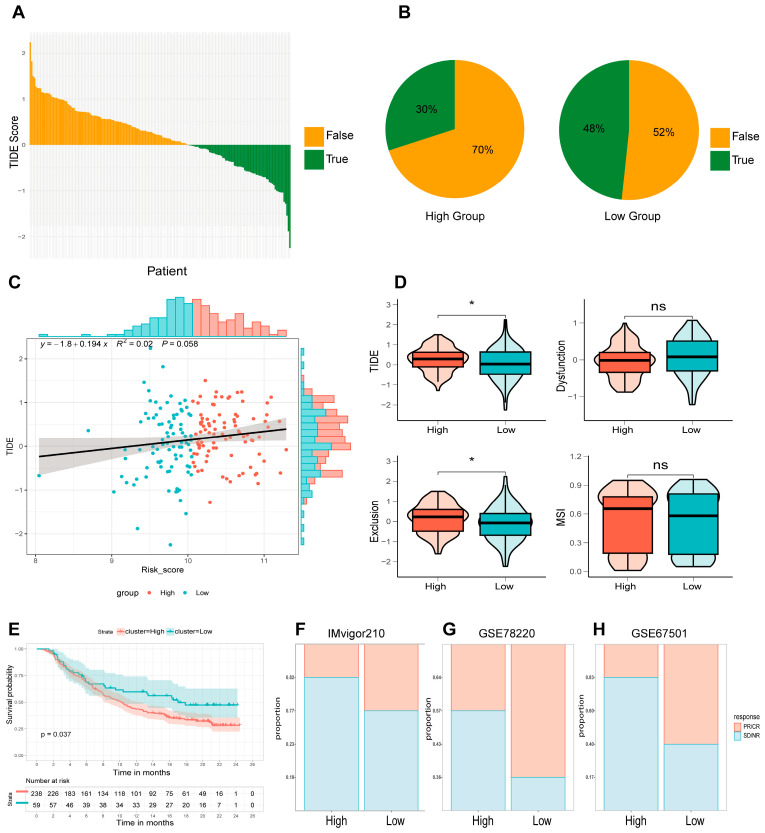
Prognostic evaluation of immunotherapeutic efficacy utilizing the 4-ARG model. (**A**) Distribution of predicted responders versus non-responders to immune checkpoint inhibitors (ICIs) within the GSE53625 cohort (*n* = 179) based on TIDE analysis. (**B**) Bar chart comparing the percentage of ICI responders between low- and high-risk groups. (**C**) Linear regression analysis correlating risk scores with TIDE scores. (**D**) Comparative analysis of TIDE, Exclusion, Dysfunction, and MSI scores across risk groups. (**E**) Kaplan–Meier survival curves for risk groups within the IMvigor210 cohort (*n* = 310). (**F**–**H**) Comparative assessment of anti-PD-L1 treatment responses between high- and low-risk groups across the IMvigor210 (*n* = 137), GSE78220 (*n* = 28), and GSE67501 (*n* = 11) datasets. (* *p* < 0.05; ns, not significant).

Given the transformative impact of immunotherapy in oncology, we employed the Tumor Immune Dysfunction and Exclusion (TIDE) framework to gauge potential responsiveness to immune checkpoint inhibitors (ICIs) within the GSE53625 dataset. We computed TIDE, exclusion, dysfunction, and microsatellite instability (MSI) scores for each patient. Using the established threshold, the algorithm distinguished responders (TIDE score < 0) from non-responders (TIDE score > 0) (Figure 6A). Notably, the low-risk group harbored a significantly higher proportion of predicted responders compared to the high-risk group (Figure 6B). Pearson analysis confirmed a positive correlation between risk and TIDE scores, suggesting that low-risk patients may derive greater benefit from ICI therapy (Figure 6C). Consistently, both TIDE and exclusion scores were significantly elevated in the high-risk cohort, suggesting stronger immune evasion and poorer response to ICIs in these patients (Figure 6D).

To further validate the predictive power of the 4-ARGs model for ICIs, we analyzed the GSE78220 and IMvigor210 datasets. In both datasets, the high-risk group exhibited a higher proportion of patients with progressive disease (PD) or stable disease (SD), indicating poorer responses to anti-PD-L1 therapy (Figure 6F–H). Importantly, the prognosis of high-risk patients was worse than that of low-risk patients in the IMvigor210 dataset (Figure 6E). These results are hypothesis-generating and require validation in ESCC-specific immunotherapy cohorts.

### 3.8. Drugs Sensitivity

Given the poorer overall survival (OS) in high-risk patients, we sought to identify potential therapeutic drugs for this group. First, we uploaded the DEGs from tumor and normal tissues in the GSE53625 dataset to the CMap database for Mechanism of Action (MoA) analysis. Figure S2A delineates the top 50 pharmacologic agents predicted to exhibit efficacy in ESCC treatment, alongside their associated signaling pathways. Notably, pathways such as topoisomerase inhibitors, mTOR inhibitors, CDK inhibitors, AKT inhibitors, HDAC inhibitors, FLT3 inhibitors, and HMGCR inhibitors exhibited a high degree of enrichment. Topoisomerase inhibitors disrupt the function of DNA topoisomerases, thereby blocking DNA replication and repair processes and inhibiting tumor cell proliferation. The discovery of novel anticancer chemotherapeutics targeting topoisomerase enzymes remains a significant focus in cancer research [50]. CDK inhibitors, which block the cell cycle by inhibiting cyclin-dependent kinases (CDKs), effectively suppress tumor cell proliferation. Specifically, CDK4/6 inhibitors, which target the enzymatic activity of CDK4 and CDK6, have been approved by the FDA for the treatment of metastatic hormone receptor-positive breast cancer [51]. These findings suggest that these drugs primarily exert their therapeutic effects in ESCC treatment by modulating tumor cell metabolism and proliferation.

“OncoPredict” analysis of the GDSC dataset identified 118 drugs exhibiting significantly different therapeutic sensitivities between high- and low-risk groups (Supplementary Table S5). An inverse correlation was detected between the risk score and the half-maximal inhibitory concentration (IC50) values for the majority of tested drugs, implying enhanced therapeutic susceptibility in high-risk patients (Figure S2B). Among the candidate genes, *NBEA* was negatively correlated with IC50 values, while *NLRX1* was positively correlated. After filtering outliers, the top 20 effective drugs were identified (Figure S3). By intersecting results from both analyses, 22 drugs were identified as significant (Figure S2C). To confirm reliability, we incorporate the Cancer Therapeutics Response Portal (CTRP v2.1), which furnishes comprehensive drug-sensitivity data across a broad spectrum of cancer cell lines, including ESCC. This analysis demonstrated that all 22 candidate drugs identified via CMap and GDSC profiling exhibit significant sensitivity in ESCC cell lines (Supplementary Table S6).

Among these, pazopanib, piperlongumine, prochlorperazine, and trifluoperazine were predicted to be particularly effective for high-risk ESCC patients, offering potential therapeutic options for this group.

### 3.9. Prognostic Value of the 4-ARGs Model and Candidate Genes in Pan-Cancer

To evaluate the pan-cancer prognostic utility of the 4-ARG signature and its constituent genes, we analyzed their associations with overall survival (OS) leveraging the TCGA database. Figure S4A presents a heatmap summarizing the hazard ratios (HRs) derived from univariate Cox regression and *p*-values from Kaplan–Meier survival assessments. The risk score was identified as a protective factor in most cancer types, with higher risk scores being associated with better prognoses. These cancers include Glioblastoma multiforme (GBM), Pancreatic adenocarcinoma (PAAD), Acute Myeloid Leukemia (LAML), Testicular Germ Cell Tumors (TGCT), Rectum adenocarcinoma (READ), Colon adenocarcinoma (COAD), Esophageal carcinoma (ESCA), Uterine Carcinosarcoma (UCS), and Kidney renal clear cell carcinoma (KIRC). Among the candidate genes, *NBEA* was protective in cancers such as thymoma (THYM), PAAD, TGCT, Mesothelioma (MESO), ESCA, UCS, and KIRC, while *CLOCK* acted as a risk factor in cancers like pheochromocytoma (PCPG), Skin Cutaneous Melanoma (SKCM), Brain Lower Grade Glioma (LGG), Thyroid carcinoma (THCA), Breast invasive carcinoma (BRCA), and Uveal Melanoma (UVM). However, *CLOCK* also served as a protective factor in LAML, READ, COAD, MESO, UCS, and KIRC. Notably, in KIRC, the risk score and all four candidate genes were significantly associated with OS, with *NBEA*, *CLOCK*, and *NLRX1* serving as protective factors and *MAGEA3* as a risk factor.

Additionally, we analyzed the expression levels of the four candidate genes in normal and tumor tissues across various cancers. The results revealed significant differences in the expression of these genes between normal and tumor tissues in multiple cancer types (Figure S4B–E). Collectively, these findings highlight the strong prognostic value of the 4-ARGs model and its candidate genes across various cancers.

### 3.10. Validation of Candidate Genes in ESCC Patients and Clinical Data Analysis

**Figure 7:**
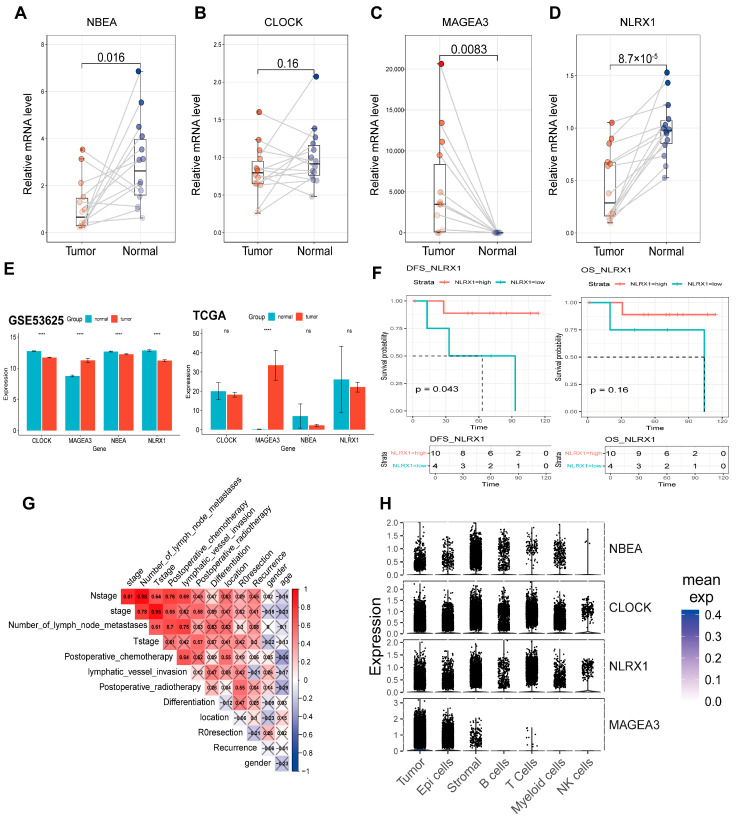
The validation of candidate genes in ESCC patients and the analysis of clinical data. (**A**–**D**) The expression of the four candidate genes in normal esophageal tissue and ESCC tissue of patients. (paired *t*-test). (**E**) The expression of the four candidate genes between normal and tumor tissue in GSE53625 (*n* = 179), TCGA-ESCC (*n* = 94); (**F**) The Kaplan–Meier survival analysis for the two groups divided by the expression of *NLRX1*; (**G**) Correlation analysis of clinical factors; (**H**) The expression of the four candidate genes in scRNA-seq data (GSE160269, *n* = 64). (**** *p* < 0.0001; ns, not significant).

To further validate the 4-ARGs model and candidate genes, we analyzed their expression in ESCC patients from the in-house cohort. *NBEA* and *NLRX1* were more highly expressed in normal tissues, while *MAGEA3* was more highly expressed in tumor tissues (Figure 7A–D). These results were consistent with findings from the two datasets and suggest that these genes may serve as therapeutic targets for ESCC (Figure 7E).

In our local validation cohort of 14 patient samples, survival analysis indicated that high-risk individuals—defined here by suppressed *NLRX1* expression—suffered from significantly reduced disease-free survival (DFS) (Figure 7F). Kaplan–Meier curves for the remaining candidate genes are provided in Figure S5. Furthermore, Gene Set Variation Analysis (GSVA) highlighted significant correlations between candidate gene expression, key autophagy markers, and autophagy scores, thereby substantiating their regulatory roles in autophagic processes (Figures S6 and S7).

Single-cell transcriptomic profiling (scRNA-seq) provided granular evidence that the candidate genes are prominently expressed in tumor, epithelial, and stromal cells. Additionally, *NBEA*, *CLOCK*, and *NLRX1* exhibited elevated expression in immune populations, including B cells, T cells, and myeloid cells. Notably, *CLOCK* and *NLRX1* also showed distinct enrichment within Natural Killer (NK) cells (Figure 7H).

The correlation analysis of clinical factors in ESCC patients from the in-house cohort was presented in Figure 7G. N stage had a significant strong positive correlation with postoperative chemotherapy and postoperative radiotherapy which revealed that postoperative chemotherapy and postoperative radiotherapy were important treatment methods for patients with a high degree of N stage. Additionally, N stage had a significant strong positive correlation with T stage and lymphatic vessel invasion, which suggested that the number of lymph node metastases was related to the size of the tumor and tumor lymphatic vessel invasion in this cohort.

Overall, in the newly collected clinical samples, the experimental results for the 4-ARGs model and its candidate genes were consistent with our bioinformatic findings, suggesting that these genes could serve as novel biomarkers for ESCC diagnosis.

## 4. Discussion

In this study, we identified four autophagy-related genes, *NBEA*, *CLOCK*, *NLRX1*, and *MAGEA3*, as prognostic factors for ESCC based on transcriptome data from the training set GSE53625. Using these four genes, we developed a prognostic risk model termed the 4-ARGs model. We substantiated the prognostic value of the 4-ARG model and its constituent genes utilizing the TCGA-ESCC dataset as an independent external cohort. The analysis confirmed the model’s capacity to effectively stratify ESCC patients based on survival probabilities, demonstrating consistent predictive performance across multiple validation metrics. Moreover, we verified the differential expression patterns of these candidate genes in clinical specimens collected from our institutional ESCC cohort. Combining patient survival data, *NLRX1* was confirmed to be a potential reliable protective prognostic factor. Gene pathway enrichment analysis revealed that autophagy and ESCC-related genes influence tumor-associated pathways such as the “Fanconi anemia pathway”, “Cell cycle” and “DNA replication” while downregulating the “Drug metabolism—cytochrome P450” and “Metabolism of xenobiotics by cytochrome P450” pathways, ultimately reducing the survival rate of ESCC patients. We also identified independent clinical prognostic factors to construct a nomogram and explored the role of candidate genes in the ESCC immune microenvironment. The TIDE algorithm was employed to confirm the 4-ARGs model’s predictive capability for patient response to immunotherapy. Finally, potential drug targets were identified, and the prognostic value of the 4-ARGs model across various cancers was explored.

Autophagy exerts a pivotal influence on the genesis and advancement of ESCC via multifaceted molecular mechanisms. Evidence indicates that the upregulation of *Drp1*, coupled with a reduction in *TFAM* protein, precipitates cytosolic mtDNA stress. This stress response subsequently activates the cGAS-STING signaling cascade, thereby triggering autophagy and fueling the progression of ESCC [52,53]. Additionally, the mitogen-activated protein kinase TAOK3 has been demonstrated to facilitate ESCC progression and cisplatin resistance by enhancing IRGM-mediated autophagy [54]. Similarly, butyrophilin subfamily 3 member A1 (BTN3A1) has been reported to promote tumor progression and radiation resistance in ESCC by regulating ULK1-mediated autophagy [55]. However, these findings are far from clinical application. The recent proliferation of high-throughput genomic technologies, alongside the establishment of comprehensive cancer expression repositories, has revolutionized our comprehension of ESCC pathology. These advancements have unveiled novel avenues for accurately prognosticating patient survival outcomes. Consequently, identifying genetic prediction models and stable, specific prognostic markers has become a crucial focus of ESCC clinical research.

Recent mechanistic studies have converged on an essential role for autophagy in sculpting the tumor microenvironment (TME). In the context of pancreatic ductal adenocarcinoma (PDAC), autophagy has been identified as a key driver of immune evasion, facilitating the lysosomal degradation of MHC-I via an *NBR1*-dependent selective mechanism [56]. Crucially, the blockade of this autophagic process restores surface MHC-I levels, effectively reversing immune evasion and synergistically augmenting the efficacy of immune checkpoint blockade therapies [56]. Parallel findings indicate that inhibiting autophagy by targeting *ULK1* re-establishes antigen presentation, thereby enhancing responses to anti-PD1 treatment [57]. Furthermore, autophagy modulates immune cell trafficking by regulating cytokine profiles within the tumor microenvironment; for instance, the ablation of *FIP200* in PyMT mammary tumors triggers the release of CXCL9/10, promoting the infiltration of cytotoxic T cells into the tumor bed [58]. Mechanistically, tumor-derived lactate suppresses FIP200, tipping the balance toward pro-apoptotic BCL2 proteins and inducing T-cell apoptosis, thereby facilitating immune evasion [59]. Collectively, these findings illustrate that autophagy acts as a rheostat that either sustains immunosuppression or, when inhibited, restores immunostimulatory signaling in the TME.

The identified candidate genes exhibit a profound association with autophagic processes. It is well-established that numerous genes governing macroautophagy operate under circadian regulation, with the Circadian Locomotor Output Cycles Kaput (*CLOCK*) gene serving as a central regulator [60]. For instance, evidence suggests that *CLOCK* modulates mitochondrial autophagy (mitophagy) and ensures cellular survival in cardiac myocytes subjected to ischemic stress [61]. Additionally, melanoma-associated antigen A3 (*MAGEA3*) has been shown to inhibit autophagy by degrading 5′ adenosine monophosphate-activated protein kinase (AMPK), with autophagy levels in patient tumors inversely correlating with MAGE expression [62]. Nucleotide-binding oligomerization domain-like receptor X1 (*NLRX1)* plays a critical role in regulating mitophagy. In the context of viral pathogenesis, *NLRX1* has been shown to suppress RIG-I-mediated IFN-1 signaling in MEFs while promoting mitophagy through its interaction with the mitochondrial matrix protein TUFM [63]. Conversely, in cisplatin-treated HEI-OC1 auditory cells, *NLRX1* overexpression amplifies ROS/JNK signaling and escalates autophagic flux, ultimately culminating in cellular injury or death. Notably, the suppression of ROS generation effectively attenuates cisplatin-induced autophagy activation [64].

The four candidate genes are directly or indirectly related to the occurrence and prognosis of ESCC, as supported by numerous studies. To provide theoretical support for this model, we discuss the potential of the candidate genes serving as stable, ESCC-specific prognostic markers. For instance, nucleotide-binding and oligomerization domain (NOD)-like receptor (NLR) proteins were initially identified as cytoplasmic pathogen recognition receptors (PRRs) critical to innate immunity. NLRX1, the only NLR known to localize to mitochondria, establishes a novel link between mitochondrial function and disease pathophysiology through its unique N-terminal domain [65]. A recent bioinformatics study revealed that *NLRX1* is expressed at low levels in ESCC compared to adjacent normal tissues, with low *NLRX1* expression correlating with shorter patient survival. Moreover, *NLRX1* was found to negatively regulate the PI3K/AKT signaling pathway in ESCC, making it an independent protective prognostic factor associated with tumor grading [66]. MAGEA3, a cancer-testis antigen (CTA), is aberrantly expressed in various cancers and has been extensively studied in cancer immunotherapy [67]. Recent findings have shown that MAGEA3 serves as an independent protective indicator for ESCC prognosis [68,69]. Additionally, anti-MAGEA3 autoantibodies, such as MAGEA3-IgG and MAGEA3-IgM, have demonstrated diagnostic potential for ESCC [70]. Reports indicate that Decitabine potentiates T-cell-mediated tumor recognition in esophageal carcinoma by upregulating *MAGEA3* expression, implying a potential tumor-suppressive role for this gene [71]. While direct evidence linking *NBEA* and *CLOCK* specifically to ESCC remains sparse, both genes have been implicated in the oncogenesis and progression of various other malignancies [72,73,74,75,76].

Our analysis revealed distinct correlations between candidate gene expression and immune cell infiltration within the tumor immune microenvironment (TIME). Specifically, high *NBEA* expression correlated with increased M0 and M2 macrophages but decreased neutrophils. Given that M2 macrophages drive immunosuppression [77] while neutrophils contribute to anti-tumor immunity [78], *NBEA* likely fosters an immunosuppressive microenvironment to promote ESCC progression. Conversely, the risk gene *CLOCK* was positively associated with M1 macrophages and activated CD4 memory T cells, yet negatively correlated with naive CD4 T cells, follicular helper T cells (Tfh), and activated mast cells. This suggests *CLOCK* may compromise adaptive immunity by disrupting T cell differentiation and reducing effector subsets. Regarding protective genes, high *NLRX1* expression paralleled increases in activated CD4 memory T cells and monocytes. By promoting cytokine secretion (e.g., IFN-γ) [79] and antigen presentation, *NLRX1* appears to enhance immune surveillance, consistent with its established role in mitophagy regulation. Finally, *MAGEA3* showed an inverse correlation with M0 macrophages, potentially limiting the precursor pool for tumor-promoting phenotypes [77]. Collectively, these findings underscore the biological plausibility of our model and elucidate the immunological mechanisms by which autophagy-related genes modulate ESCC progression.

With an AUC exceeding 0.7, our 4-ARG model demonstrated superior predictive accuracy compared to several previously established ESCC prognostic signatures. These include the Autophagy-Related Three-Gene Prognostic Signature [80], a fibroblast-associated signature [81], an RNA modification-related prognostic signature [82], and a ferroptosis/iron-metabolism signature [83] (Figure S8).

Notwithstanding these promising results, this study is subject to certain limitations. Primarily, the reliance on relatively limited public databases may restrict the model’s universal applicability. Furthermore, additional investigations are requisite to fully validate the clinical utility and underlying biological mechanisms of this autophagy-related signature. Future endeavors should aim to incorporate larger cohorts, integrate comprehensive clinical-pathological parameters, and conduct extensive experimental studies to mechanistically elucidate the functions of these key genes. The construction of the 4-ARG prognostic model relied on stepwise multivariate Cox regression. While this is a standard variable selection method, it is known to be sensitive to data perturbations and sampling variability, which may influence the stability of the selected features. Additionally, we acknowledge that a formal assessment of collinearity among the clinical variables included in the nomogram was not conducted. While variables were selected based on clinical availability and statistical significance, potential inter-correlations between factors such as tumor location and stage cannot be fully ruled out. Finally, although we performed experimental validation using qRT-PCR to corroborate our bioinformatics findings, the statistical power of this analysis was constrained by the relatively small sample size (*n* = 14) of our in-house cohort. Therefore, the protective role of *NLRX1* observed in clinical samples should be considered preliminary, and large-scale, multi-center prospective validation is essential to definitively establish the clinical utility of these biomarkers. This will provide a solid foundation for advancing ESCC treatment and developing molecular targets.

## 5. Conclusions

In summary, we established a robust 4-gene autophagy-related signature (*NBEA*, *CLOCK*, *NLRX1*, and *MAGEA3*) that serves as an independent prognostic factor for ESCC. Beyond risk stratification, our study elucidates the crosstalk between autophagy and the tumor immune microenvironment, offering hypothesis-generating insights into immunotherapy responsiveness. Furthermore, we identified candidate drugs like pazopanib for high-risk patients and preliminarily validated *NLRX1* as a protective biomarker. Collectively, this model provides a valuable tool for personalized prognostic assessment and therapeutic optimization in ESCC.

## Data Availability

The original data presented in the study are openly available in the Gene Expression Omnibus (GEO) database (https://www.ncbi.nlm.nih.gov/geo, accessed on 8 December 2024) and The Cancer Genome Atlas (TCGA) database (https://www.cancer.gov/tcga/, accessed on 8 December 2024).

## Supplementary Materials

The following supporting information can be downloaded at: https://www.mdpi.com/article/10.3390/cancers18030388/s1, Figure S1: The Kaplan–Meier survival analysis curves stratified by high and low groups based on the expression levels of four candidate genes in GSE53625 (*n* = 358) (**A**), TCGA-ESCC (*n* = 94) (**B**); Figure S2: Drug sensitivity analysis (GSE53625, *n* = 179). (**A**) The top 50 drugs predicted by CMap to be most effective for the treatment of ESCC, along with their associated pathways; (**B**) Correlation between the 4-ARGs model and the IC50 value of the 118 drugs with significant therapeutic effect differences between high- and low-risk groups predicted by Oncopredict; (**C**) The intersection of the drugs identified in both sensitivity analyses; Figure S3: The top 20 effective drugs of the 118 drugs with significant therapeutic effect differences between high- and low-risk groups predicted by Oncopredict (removing outliers); Figure S4: The prognostic value of the 4-ARGs model and candidate genes in pan-cancer. (**A**) The OS heatmap presenting the Hazard Ratio (HR) of the univariate Cox regression based on risk score and candidate genes expression and *p*-values of the KM curves (removing cancer with HR > 5); (**B**–**E**) Comparison of the candidate genes expression in normal and tumor tissue in pan-cancer; Figure S5: The Kaplan–Meier survival analysis curves stratified by high and low groups based on the expression levels of four candidate genes in the clinical data collected from 14 ESCC patients; Figure S6: Linear regression analysis between autophagy score and the expression of the four candidate genes; Figure S7: Linear regression analysis between autophagy score and the expression of the ATG5 in GSE53625; Figure S8: The ROC curves of the 4-ARGs model and other four previously published prognostic signatures for ESCC in GSE53625; Table S1: An autophagy-related gene set containing 1357 genes collected from HADb, MSigDB, HAMdb, ncRDeathDB database, Autophagy Database; Table S2: The summary of all datasets [15,16,17,18,19,20,21,22,23]; Table S3: The clinical information of patients in the training set (GSE53625) [19], validation set (TCGA-ESCC) and qPCR validation cohort; Table S4: The primer sequences of *NBEA*, *CLOCK*, *NLRX1* and *MAGEA3*; Table S5: The IC50 value of 118 drugs from the GDSC database that showed significant differences in therapeutic effects between the two groups; Table S6: The significant sensitivity of 22 candidate drugs identified through CMap and GDSC in ESCC cell lines.

## Abbreviations

The following abbreviations are used in this manuscript:

ESCC	Esophageal Squamous Cell Carcinoma
ARG	Autophagy-Related Gene
DEG	Differentially Expressed Gene
OS	Overall Survival
DFS	Disease-Free Survival
HR	Hazard Ratio
AUC	Area Under the Curve
ROC	Receiver Operating Characteristic
KM	Kaplan–Meier
TIME	Tumor Immune Microenvironment
ICI	Immune Checkpoint Inhibitor
CR	Complete Response
PR	Partial Response
PD	Progressive Disease
SD	Stable Disease
IC50	Half Maximal Inhibitory Concentration
qRT-PCR	Quantitative Reverse Transcription Polymerase Chain Reaction
NAT	Normal Adjacent Tissue
FDR	False Discovery Rate
NES	Normalized Enrichment Score
TME	Tumor Microenvironment
