# Supplementary material for: Identification and Validation of an Autophagy-Related Gene Signature for Prognostic Prediction and Immunotherapy Response in Esophageal Squamous Cell Carcinoma
Source: Cancers (Basel). 2026 Jan 27;18(3):388. doi: 10.3390/cancers18030388 (PMC12897147; doi:10.3390/cancers18030388)
Supplement: Supplementary file 1 [file cancers-18-00388-s001.zip › suplementary table 3.pdf]

Supplementary Table 3. The clinical information of patients in the validation set (GSE53625), training set (TCGA-ESCC) and qPCR validation cohort.

Variable		GSE53625 [1]	TCGA_ESCC	PCR cohort
Total	N	179	94	14
Age	Median (y)	59.65205	57	60
	Mean (y)	59.34597	58.40426	60.79871
Gender	Female	33 (18.44%)	14 (14.89%)	4 (28.47%)
	Male	146 (81.56%)	80 (85.11%)	10 (71.43%)
TNM stage	Stage I	10 (5.6%)	7 (7.61%)	5 (35.71%)
	Stage II	77 (43.01%)	42 (45.65%)	2 (14.29%)
	Stage III	92 (51.39%)	39 (42.39%)	6 (42.86%)
	Stage IV	0	4 (4.35%)	1 (7.14%)
adjuvant therapy	yes	104 (58.10%)	1 (1.67%)	4 (28.57%)
	no	45(25,14%)	45 (75%)	10 (71.43%)
	unkown	30 (16.76%)	14 (23.22%)	0
radiation therapy or treatment	yes	/	45 (47.87%)	5 (35.71%)
	no	/	39 (41.48%)	9 (64.29%)
	unkown	/	10(10.65%)	0
race	White	0	41	0
	Asian	179	45	14
	Black or african american	0	5	0
	Unknown	0	3	0
status	Alive	73 (40.78%)	63 (67.02%)	11 (78.57%)
	Dead	106 (59.22%)	31 (32.98%)	3 (21.43%)
Survival time	Mean (m)	34.66667	12.81667	48.84551

Reference:

1. Li, J.; Chen, Z.; Tian, L.; Zhou, C.; He, M. Y.; Gao, Y.; Wang, S.; Zhou, F.; Shi, S.; Feng, X.; et al. LncRNA profile study reveals a three-lncRNA signature associated with the survival of patients with oesophageal squamous cell carcinoma. *Gut* **2014**, *63* (11), 1700-1710. DOI: 10.1136/gutjnl-2013-305806 From NLM.
