# Supplementary figures and images for: Identification and Validation of an Autophagy-Related Gene Signature for Prognostic Prediction and Immunotherapy Response in Esophageal Squamous Cell Carcinoma
Source: Cancers (Basel). 2026 Jan 27;18(3):388. doi: 10.3390/cancers18030388 (PMC12897147; doi:10.3390/cancers18030388)

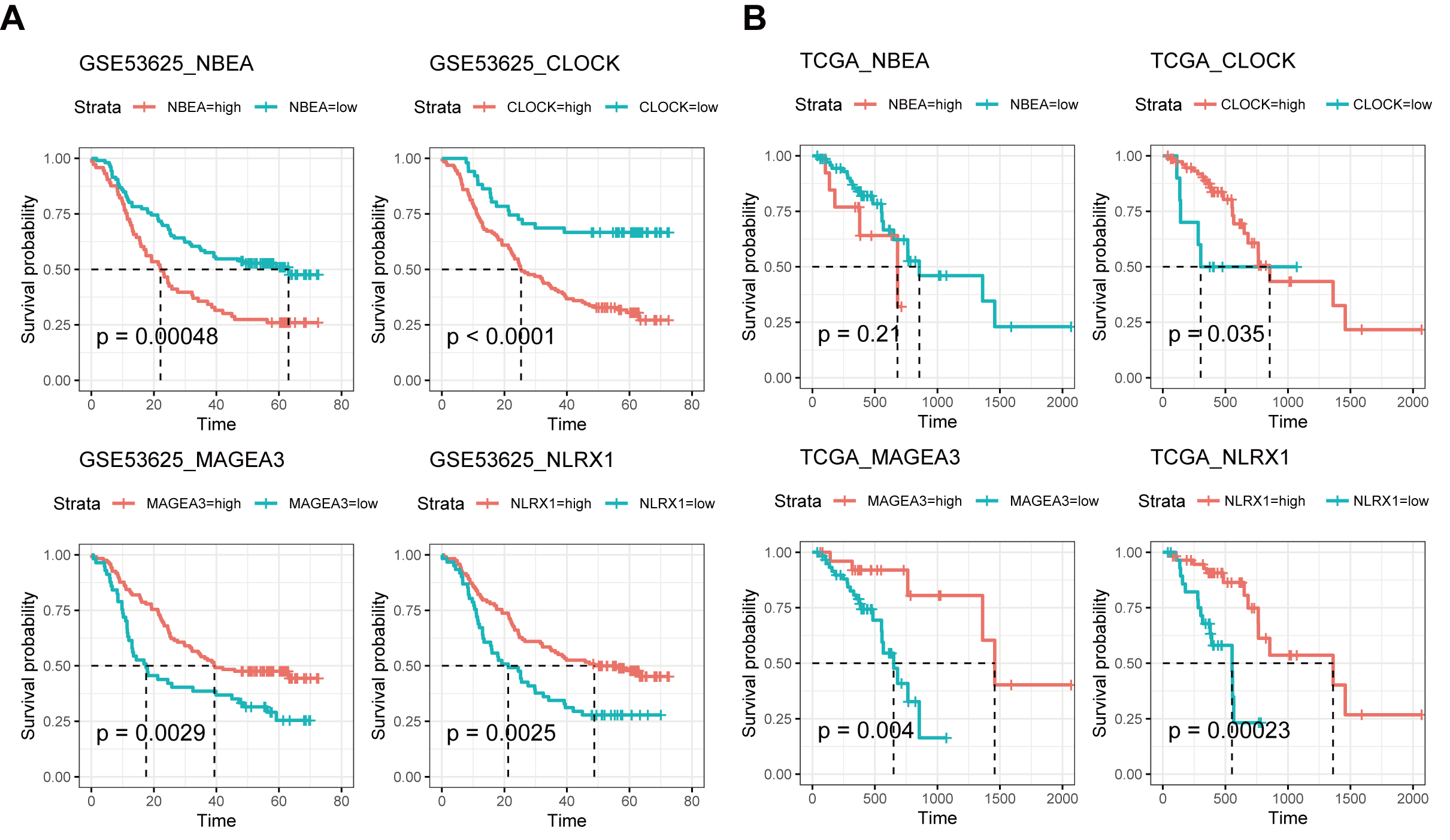

Supplement: Supplementary file 1 [file cancers-18-00388-s001.zip › Figure S1.tif]

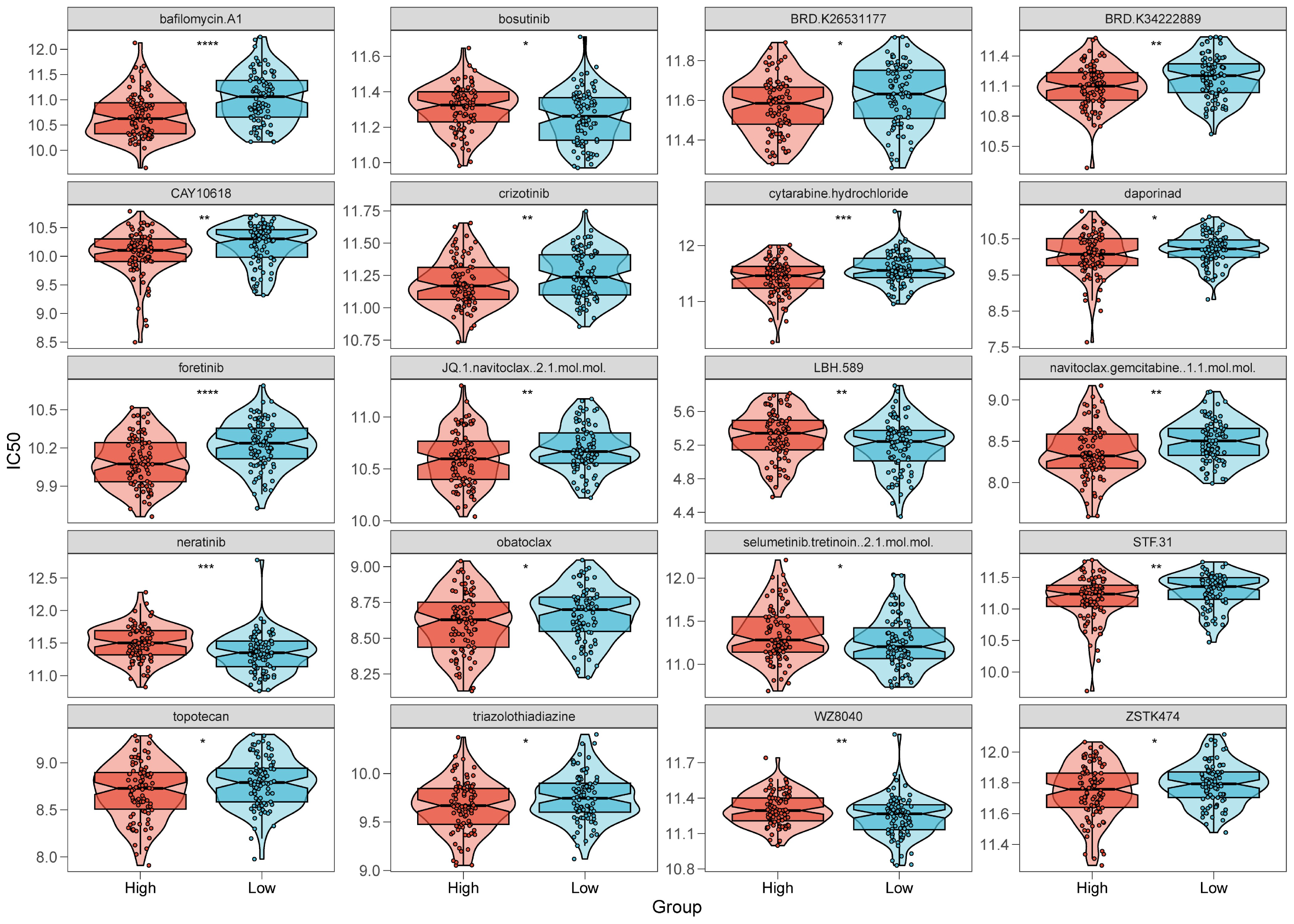

Supplement: Supplementary file 1 [file cancers-18-00388-s001.zip › Figure S3.tif]

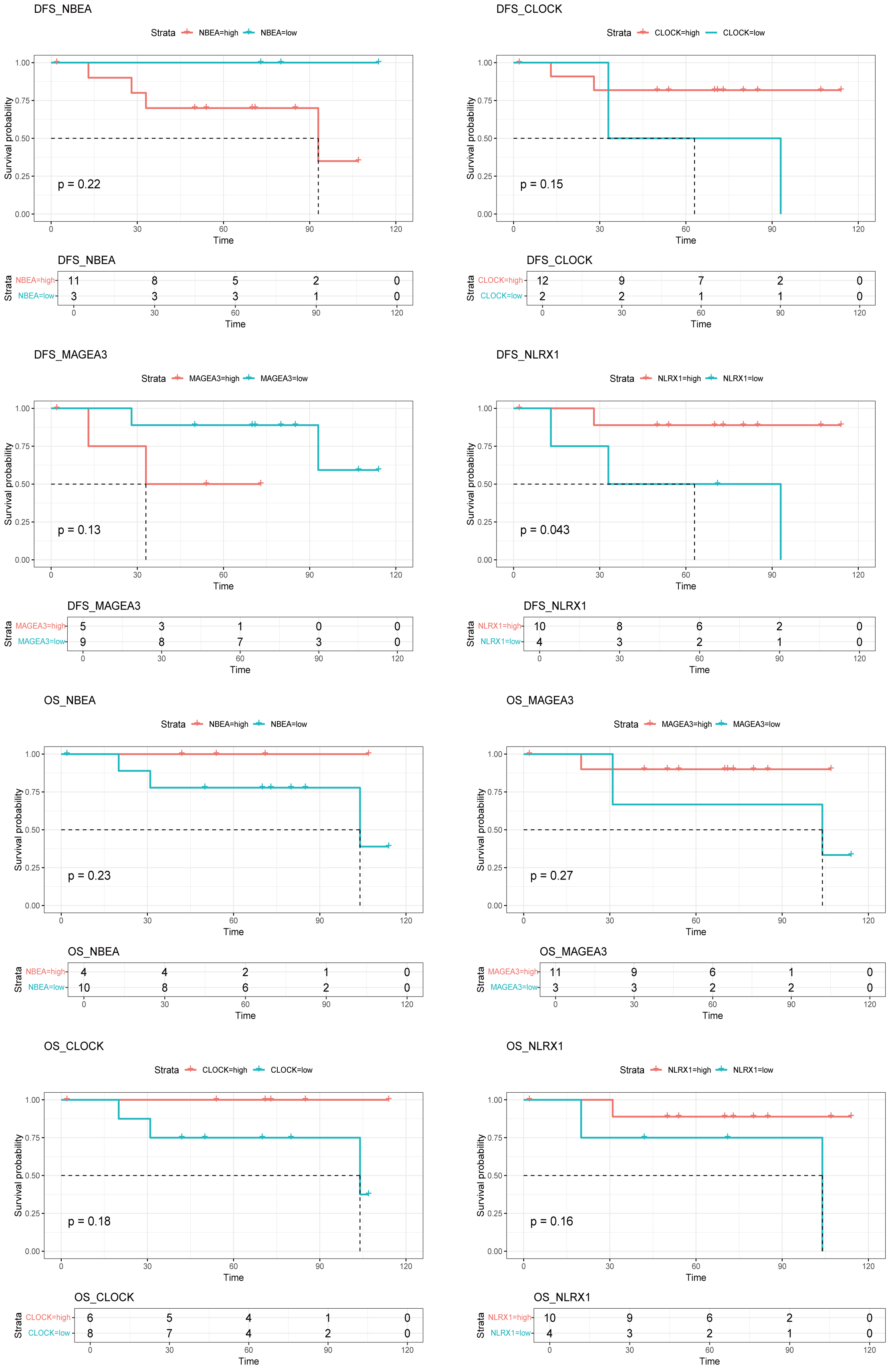

Supplement: Supplementary file 1 [file cancers-18-00388-s001.zip › Figure S5.tif]

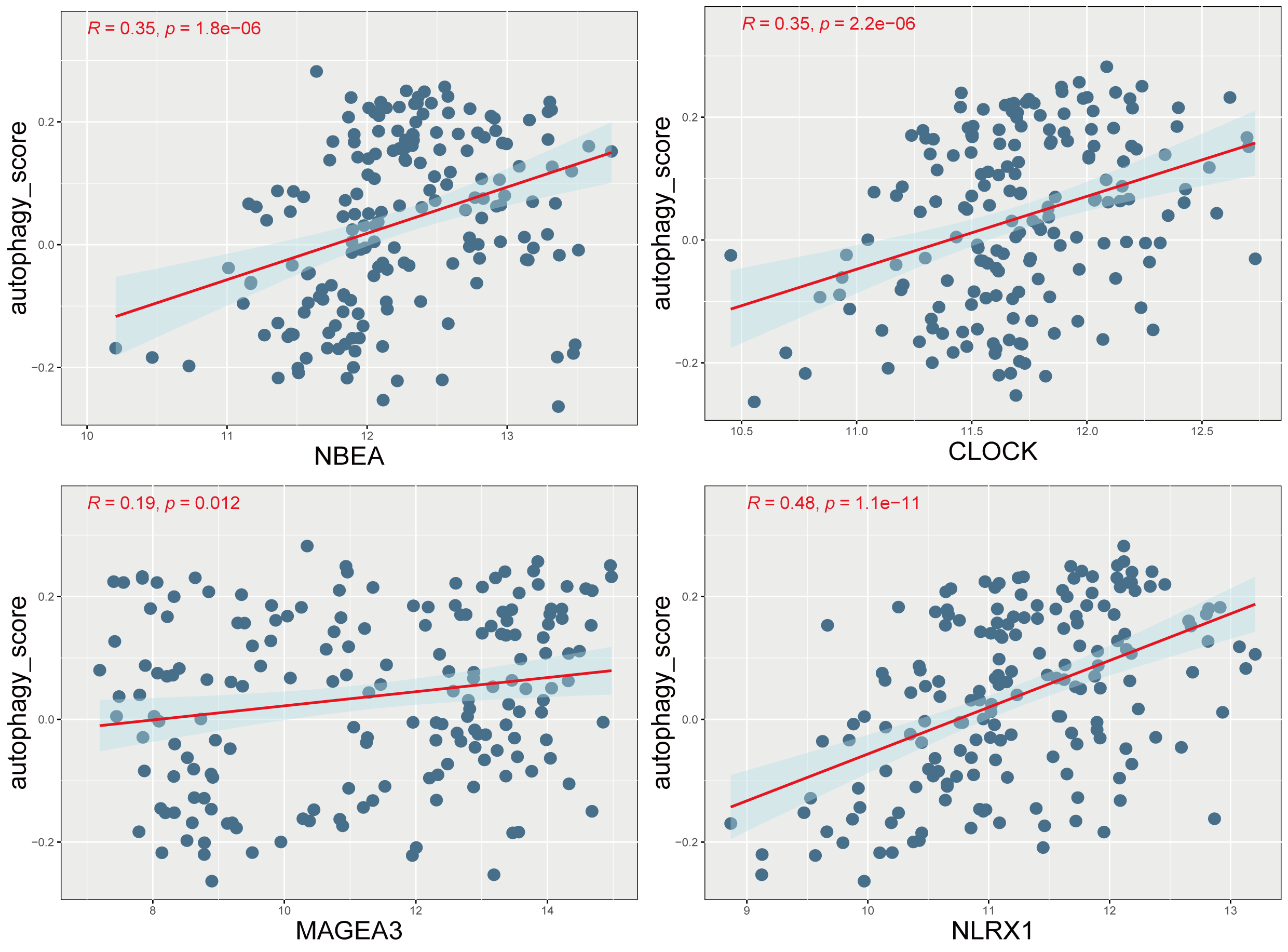

Supplement: Supplementary file 1 [file cancers-18-00388-s001.zip › Figure S6.tif]

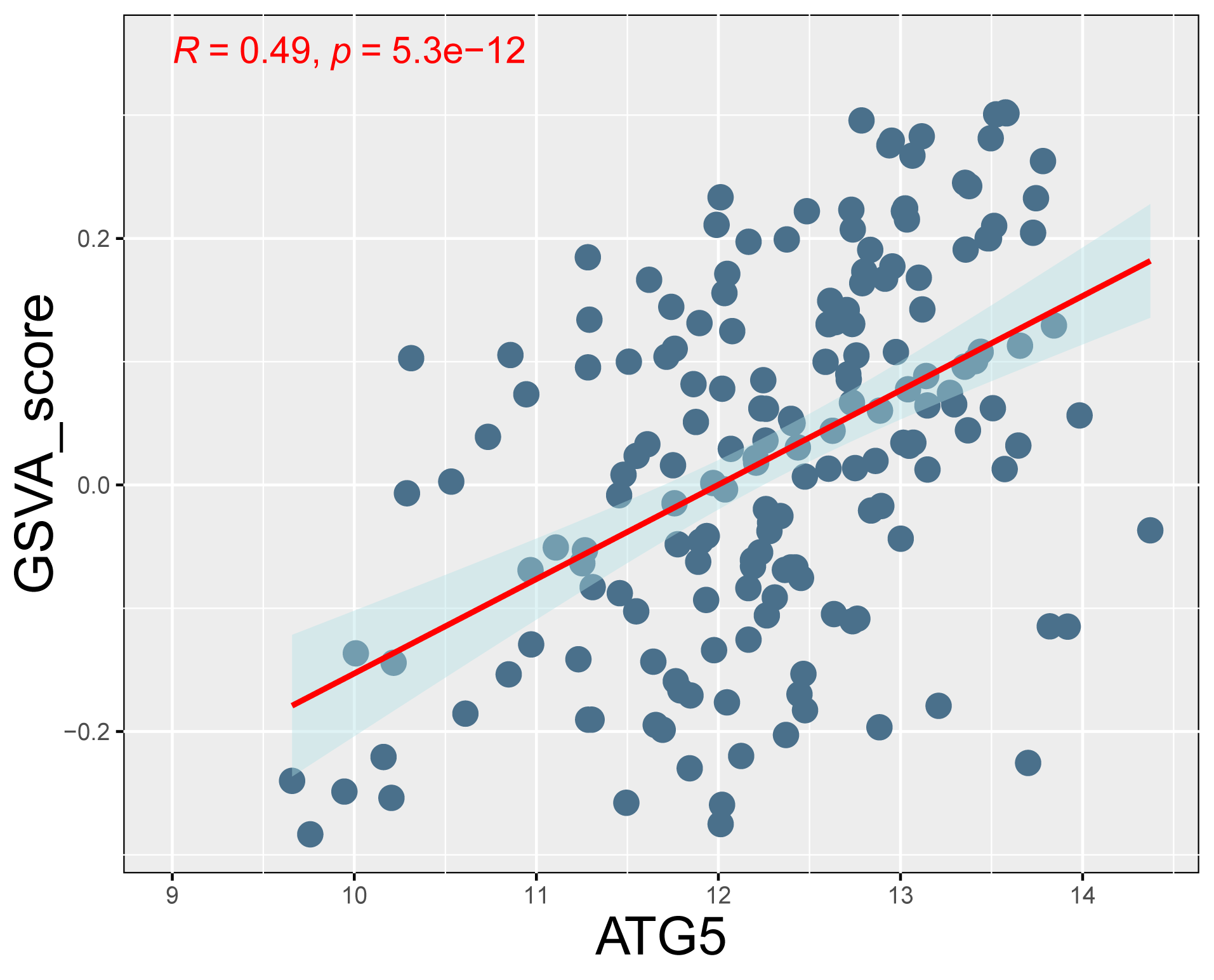

Supplement: Supplementary file 1 [file cancers-18-00388-s001.zip › Figure S7.tif]

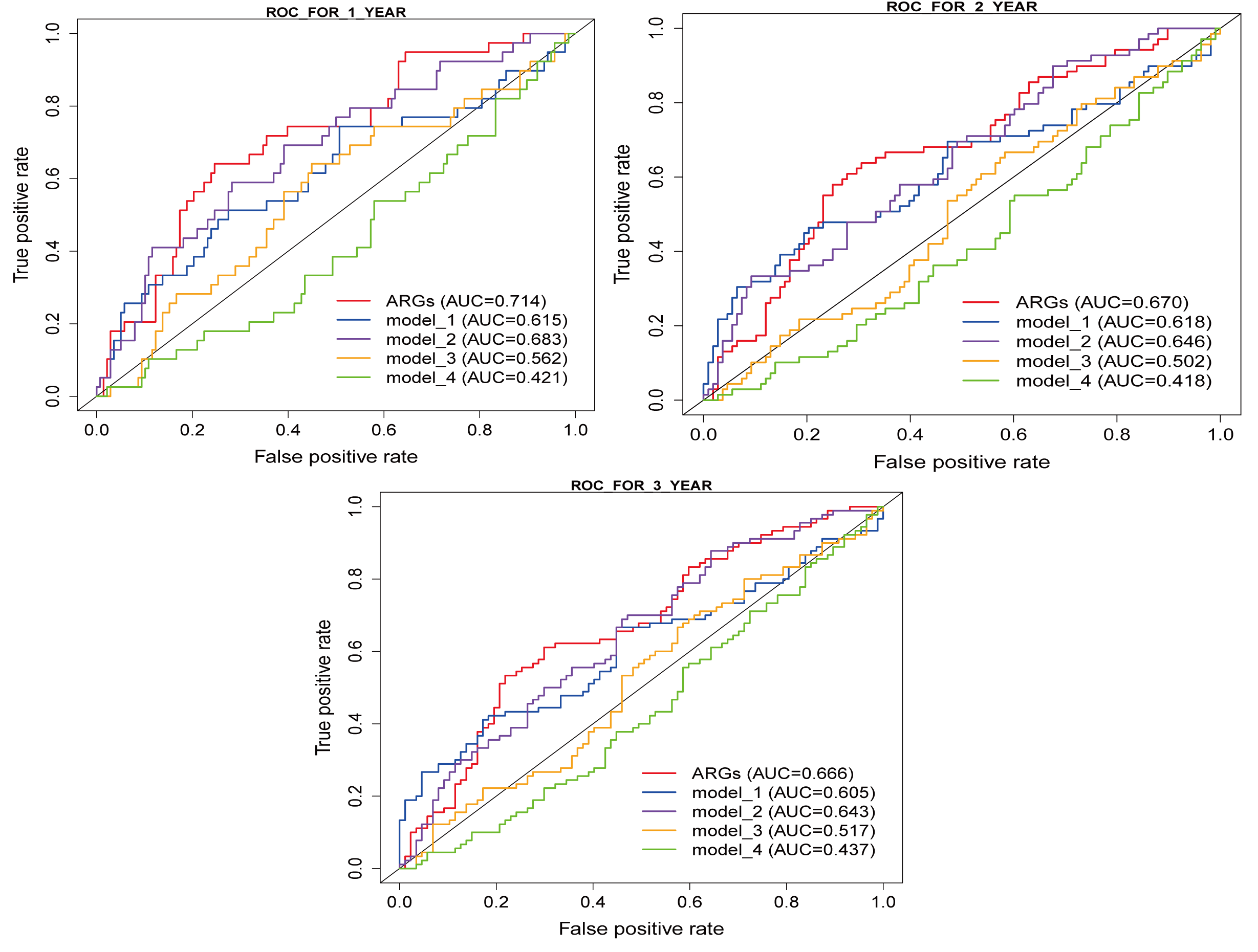

Supplement: Supplementary file 1 [file cancers-18-00388-s001.zip › Figure S8.tif]
